# Persistent ferromagnetic ground state in pristine and Ni-doped Fe_3_GaTe_2_ flakes

**DOI:** 10.1186/s40580-024-00458-x

**Published:** 2024-12-12

**Authors:** Ki-Hoon Son, Sehoon Oh, Junho Lee, Sobin Yun, Yunseo Shin, Shaohua Yan, Chaun Jang, Hong-Sub Lee, Hechang Lei, Se Young Park, Hyejin Ryu

**Affiliations:** 1 Center for Semiconductor Technology, Korea Institute of Science and Technology (KIST), Seoul, 02792 South Korea; 2 Department of Advanced Materials Engineering for Information and Electronics, Kyung Hee University, Yongin, 17104 South Korea; 3 Department of Physics and Origin of Matter and Evolution of Galaxies (OMEG) Institute, Soongsil University, Seoul, 06978 South Korea; 4 Integrative Institute of Basic Sciences, Soongsil University, Seoul, 06978 South Korea; 5 School of Physics and Beiing Key Laboratory of Optoelectronic Functional Materials MicroNano Devices, Renmin University of China, Beijing, 100872 China; 6 Key Laboratory of Quantum State Construction and Manipulation (Ministry of Education), Renmin University of China, Beijing, 100872 China

**Keywords:** Room-temperature van der waals ferromagnet, Fe_3_GaTe_2_, Ni doped Fe_3_GaTe_2_, Magnetism, Thin film device application

## Abstract

Room-temperature magnetism and its stability upon miniaturization are essential characteristics required for materials for spintronic devices and information storage. Among various candidates, Fe_3_GaTe_2_ stands out due to its high Curie temperature and strong perpendicular magnetic anisotropy (PMA), recently gaining large attention as one of the promising candidate materials for spintronics applications. In this study, we measured the thickness-dependent ferromagnetic properties of Fe_3_GaTe_2_ and (Fe_1 − x_Ni_x_)_3_GaTe_2_ (with x = 0.1) flakes. We observed that both pristine and Ni-doped Fe_3_GaTe_2_ exhibit persistent ferromagnetism, with only a minor decrease in T_C_ as the thickness is reduced to a few tens of nanometers. This capacity to retain robust ferromagnetic properties at reduced dimensions is highly advantageous for thin-film applications, which is crucial for the scaling of spintronic devices. Understanding and controlling thickness-dependent magnetic properties is fundamental to harnessing the full potential of Fe_3_GaTe_2_ in van der Waals magnetic heterostructures and advanced spintronic technologies.

**Graphical Abstract:**

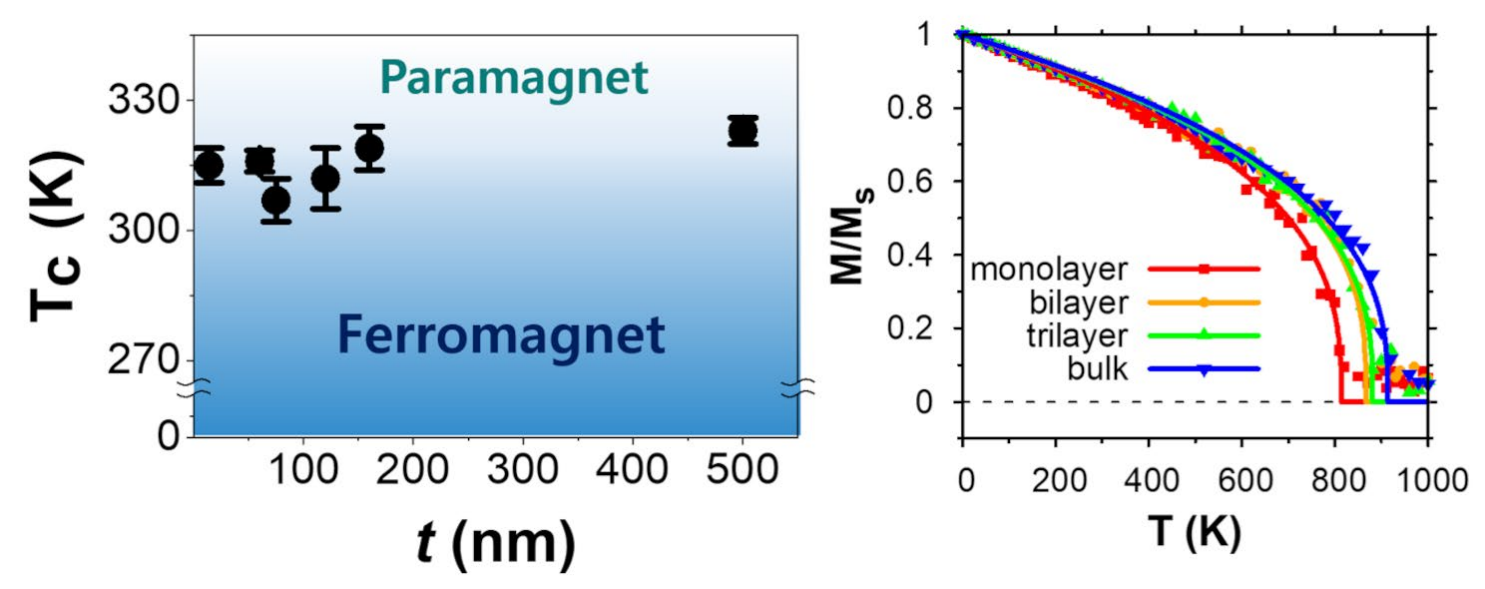

**Supplementary Information:**

The online version contains supplementary material available at 10.1186/s40580-024-00458-x.

## Introduction

Recent achievements in essential technologies such as artificial intelligence (AI), augmented reality (AR), the Internet of Things (IoT), and deep learning have led to a dramatic increase in data processing demands [1, 2]. This escalation in information throughput has necessitated significant improvements in hardware to thermal management and efficiency challenges. Conventional memory technologies face limitations in meeting these demands, prompting exploration into alternative semiconductor technologies such as resistive random-access memory (ReRAM), phase-change random-access memory (PCRAM), high bandwidth memory (HBM), compute express link (CXL), processing in memory (PIM) and spintronics [3, 4]. These technologies, excluding spintronics, operate based on the movement of charge carriers and ions. However, spintronics exploits the intrinsic spin of electrons to process information, offering the potential to reduce energy consumption and achieve higher processing speeds compared to traditional charge-based electronic devices [5–7]. For spintronic devices to effectively store information, it is necessary to exhibit ferromagnetic properties [1, 8]. Ferromagnetism ensures that the electron spins within the material align uniformly. This alignment can be controlled to define the on/off states of the devices.

VdW materials are extensively studied due to their remarkable properties, such as bandgap engineering, charge density waves (CDW), optical responses, thermoelectric properties, superconductivity, high electron mobility, and ferromagnetism [9–20]. Among these, the key characteristic of spintronics is ferromagnetism, combined with magnetic anisotropy by spin-orbit coupling, stabilizing long-range magnetic ordering and changing the direction of magnetization [21–23]. Recent studies have focused on vdW materials exhibiting ferromagnetism, including CrI_3_, CrSiTe_3_, CrGeTe_3_, MnSe_2_, and Fe_n_GeTe_2_ [22, 24–29]. Maintaining ferromagnetic properties at room temperature is essential for application in spintronic devices, yet materials with these characteristics are relatively scarce. Among these, Fe_n_GeTe_2_ (with *n* = 3, 4, 5) has emerged as a strong candidate for spintronic applications due to its tunable magnetic properties and relatively high Curie temperature (T_C_) [28, 29]. Additionally, recent studies have demonstrated that Fe_3_GaTe_2_, synthesized by substituting Ge with Ga, retains ferromagnetic behavior at temperatures above room temperature with strong perpendicular magnetic anisotropy (PMA), making it advantageous for spintronic devices [30–33].

The ability to precisely modulate the magnetic properties of vdW materials through thickness variation and doping is essential for advancing spintronic and quantum technologies. Thickness adjustments enable precise control over magnetic characteristics such as anisotropy and T_C_ while doping further influences these properties by modifying the material’s electronic and magnetic interactions. In vdW ferromagnets, a reduction in thickness generally leads to a weakening of ferromagnetism, accompanied by a decrease in both transition temperature and coercivity [28]. This poses a significant challenge for thin-film spintronic applications, necessitating the identification of materials that can maintain a robust ferromagnetic ground state despite reduced dimensionality.

In this work, we investigate the thickness dependence magnetic properties of Fe_3_GaTe_2_ and (Fe_1 − x_Ni_x_)_3_GaTe_2_ (with x = 0.1) by magneto-optical Kerr effect (MOKE) measurements and density functional theory (DFT) calculations. Our study revealed that the T_C_ for both Fe_3_GaTe_2_ and (Fe_1 − x_Ni_x_)_3_GaTe_2_ (with x = 0.1) persist with only a slight decrease as the thickness is reduced to approximately 14 nm and 50 nm, respectively, consistent with our DFT calculation results. This stability is advantageous for thin-film device applications, as ferromagnetic properties can be preserved even at thicknesses down to a few tens of nanometers—a critical requirement for device scaling in spintronics.

## Results and discussions

### Dimensionality effect of magnetic properties of Fe_3_GaTe_2_

**Fig. 1:**
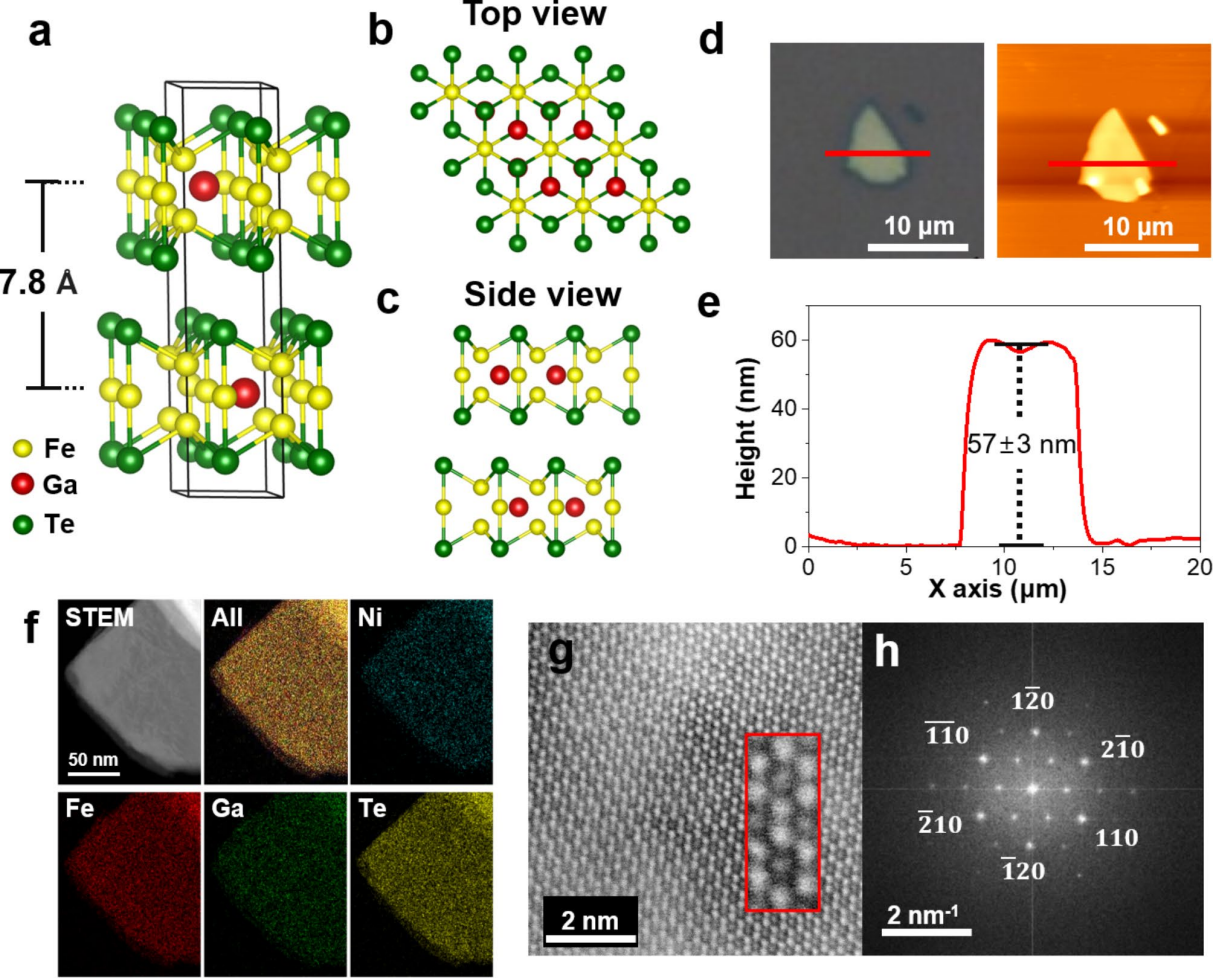
Crystal characterization of the Fe_3_GaTe_2_and (Fe_1 − x_Ni_x_)_3_GaTe_2_(with x = 0.10) single crystals and flakes. **a**. Crystal structure of Fe_3_GaTe_2_ with unit cell (black solid lines). Top view (**b**) and side view (**c**) of the atomic configurations. **d**. Optical microscope (left) and AFM (right) image of Fe_3_GaTe_2_ flake on SiO_2_/Si substrate. **e**. Height profile of Fe_3_GaTe_2_ flake measured by AFM along the red line in (d). **f.** STEM image of a (Fe_1 − x_Ni_x_)_3_GaTe_2_ (with x = 0.10) flake with corresponding EDS mapping for all, Ni, Fe, Ga, and Te elements. **g.** High-magnification STEM image of (Fe_1 − x_Ni_x_)_3_GaTe_2_ (with x = 0.10), with a magnified view of 2-unit cell shown in the red-outlined inset. **h.** FFT data for (Fe_1 − x_Ni_x_)_3_GaTe_2_ (with x = 0.10)

The Fe_3_GaTe_2_ single crystal has a hexagonal structure with a space group of P6_3_/mmc, characterized by lattice parameters *a* = *b* = 4.09(2) Å and *c* = 16.07(2) Å (Fig. 1a-c). Fe_3_GaTe_2_ flakes were mechanically exfoliated onto a SiO_2_/Si substrate using scotch tape (Fig. 1d), and atomic force microscopy (AFM) height profiles confirmed the presence of flat surfaces, enabling the identification of flake thickness (Fig. 1e).

The high-resolution TEM images (Fig. 1f) and FFT results (Fig. 1h) clearly demonstrate the single-crystal nature of Ni-doped Fe_3_GaTe_2_, consistent with the structural characteristics of pristine Fe_3_GaTe_2_, as reported previously [34]. Additionally, STEM analysis (Fig. 1f) confirms that Ni is homogeneously distributed throughout the sample. To further assess any structural changes from Ni doping, we performed single-crystal XRD analysis. The single crystal XRD results for Ni-doped Fe_3_GaTe_2_ confirm it retains the hexagonal structure with a space group of P6_3_/mmc, identical to pristine Fe_3_GaTe_2_. The lattice parameters obtained from single-crystal XRD are as follows: Fe_3_GaTe_2_ (a = b = 4.070(2) Å, c = 16.222(13) Å) and Ni-doped Fe_3_GaTe_2_ (a = b = 4.058(3) Å, c = 16.08(2) Å), consistent with the previous report [35]. These results confirm that Ni-doped Fe_3_GaTe_2_ is a single crystal, with no structural changes from doping aside from minor lattice parameter adjustments, indicating successful Ni doping.

**Fig. 2:**
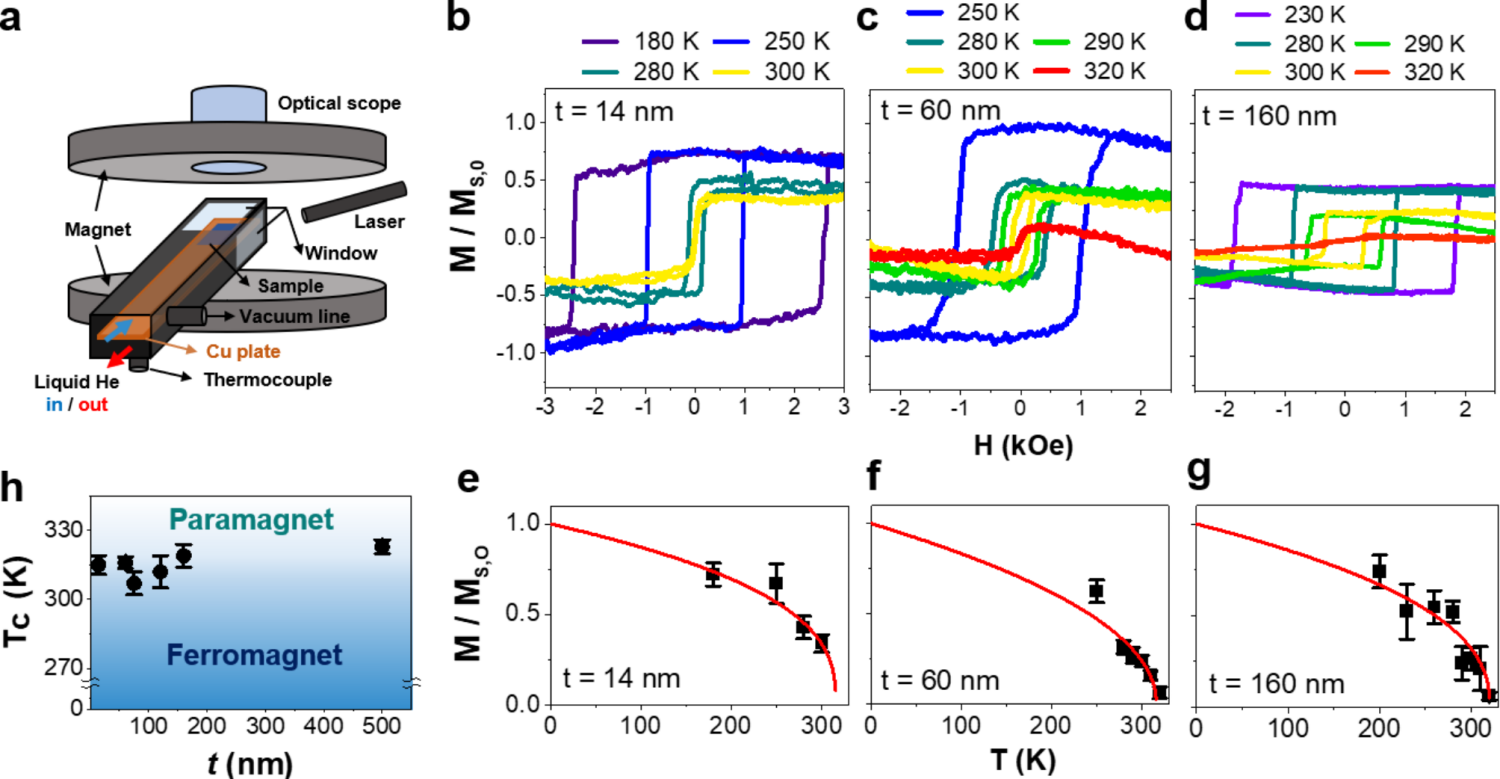
Thickness dependence of magnetic properties of pristine Fe_3_GaTe_2_. **a.** Schematic image of MOKE system. **b-d.** Temperature-dependent out-of-plane M-H loops of Fe_3_GaTe_2_ flakes measured by MOKE system at several thicknesses (t). (M-H loops for other thickness are shown in the supplementary materials (SM), Figure S2). The colors uniformly represent each temperature. **e**-**f**. Temperature-dependent Ms extracted from **b**-**d** along with the fitting curves (red solid lines). The magnetizations in **b**-**g** are normalized by the extrapolated M_S_ at T = 0 (M_S,0_) obtained from the fitting in **e**-**g**. **h**. Thickness dependence of T_C_ derived from the M-T data in **e**-**g** and Figure S2 in SM

Figure 2a displays a schematic diagram of our magneto-optical Kerr effect measurement system, which utilizes liquid helium and a magnet oriented perpendicular to the sample plane. The temperature-dependent out-of-plane magnetic hysteresis loops of Fe_3_GaTe_2_ for various thicknesses are illustrated in Fig. 2b-d and Figure S2 in supplementary materials (SM). Up to 300 K, the M-H loops exhibit a square shape, indicating relatively high remanence, which gradually decreases and approaches zero at higher temperatures. This behavior confirms that Fe_3_GaTe_2_ possesses PMA, demonstrating the ferromagnetic properties essential for spintronics applications [33]. Magnetic coercivities also decrease with increasing temperature and nearly vanish as temperature approaches T_C_, which is typical behavior for magnetic systems nearing the 2D limit.

As temperature increases, the saturation magnetization (M_S_) decreases, eventually vanishing entirely at a temperature around 310–320 K, indicating a ferromagnetic to paramagnetic phase transition and suggesting T_C_ around 310–320 K for flakes with *t* = 14–500 nm (Fig. 2e-h and Figure S2 in SM). The T_C_ for each exfoliated flake can be determined by fitting the *M*-*T* data (red solid lines in Fig. 2e-g and Figure S2 in SM) using the Eq. 

1 $$\:m\propto\:{\left(\frac{{T}_{c}-T}{{T}_{c}}\right)}^{\beta\:},$$

where $m( \equiv \:\frac{{{M_S}\left( T \right)}}{{{M_S}(T = 0)}})$ is the normalized magnetization, and *β* is the critical exponent. The *T*_c_ values for the 14 nm, 60 nm, and 160 nm Fe_3_GaTe_2_ flakes, obtained by fitting the data in Fig. 2e-g using Eq. (1), are 315 K, 316 K, and 319 K, respectively. As the thickness decreases from bulk to 14 nm, the Tc only exhibits a slight reduction of approximately 10%, confirming the persistence of the ferromagnetic state in Fe_3_GaTe_2_. The retention of a T_C_ above room temperature, even in flakes as thin as a few tens of nanometers, underscore Fe_3_GaTe_2_’s suitability as an ideal candidate for thin-film-based spintronic devices. The average critical exponent for the Fe_3_GaTe_2_ flakes is *β* = 0.327, indicating that the flakes remain in the three-dimensional ferromagnetism regime [29].

### Dimensionality effect of magnetic properties of (Fe_1 − x_Ni_x_)_3_GaTe_2_ (with x = 0.1)

**Fig. 3:**
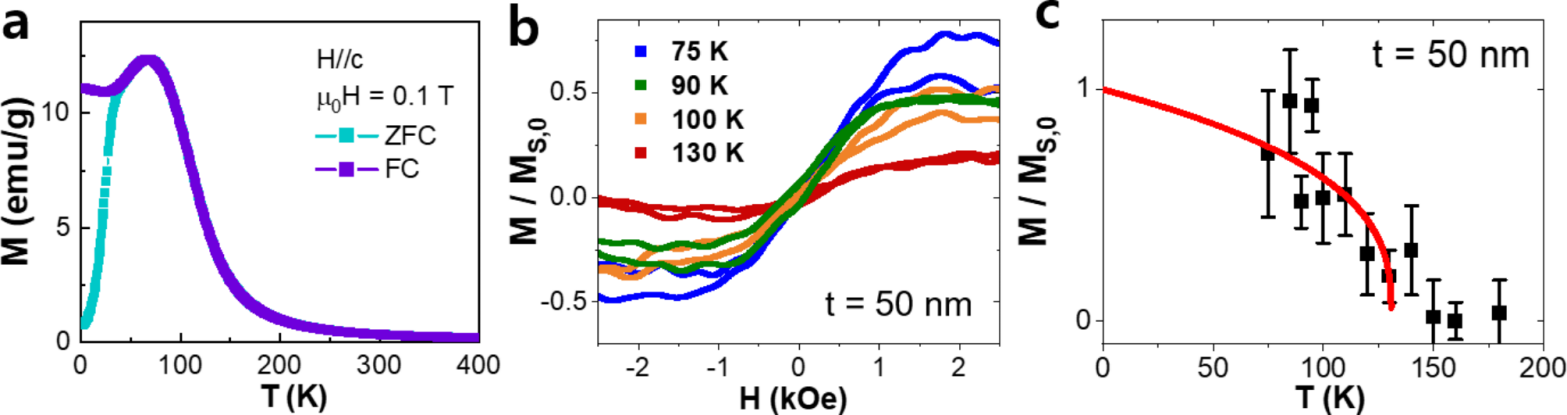
Thickness dependence of magnetic properties of (Fe_1 − x_Ni_x_)_3_GaTe_2_(with x = 0.1). **a**. Temperature-dependent out-of-plane magnetization of bulk single-crystal (Fe_1 − x_Ni_x_)_3_GaTe_2_ (with x = 0.1), measured under zero-field cooling (ZFC) and field cooling (FC) with µ_0_H = 0.1 T. **b**. Temperature-dependent out-of-plane magnetic hysteresis loops for a (Fe_1 − x_Ni_x_)_3_GaTe_2_ (with x = 0.1) flake with a thickness (t) of 50 nm. **c**. Temperature-dependent M_S_ for the 50 nm thick (Fe_1 − x_Ni_x_)_3_GaTe_2_ (with x = 0.1) flake derived from **b**, along with the fitting curve (red solid line). The magnetizations in **b** and **c** are normalized by the extrapolated M_S_ at T = 0 (M_S,0_) obtained from the fitting in **c**

To explore the dimensionality effects in (Fe_1 − x_Ni_x_)_3_GaTe_2_ (with x = 0.10), we conducted magnetization measurements on both a bulk single crystal (Fig. 3a) and a 50 nm thick flake (Fig. 3b, c). The Fe_3_GaTe_2_ single crystal maintains its ferromagnetic ground state with 10% Ni doping, exhibiting typical ferromagnetic behavior (Fig. 3a). Ni is nonmagnetic in this structure [36, 37], which suppresses the ferromagnetic properties, leading to a reduction in the T_C_ of (Fe_1 − x_Ni_x_)_3_GaTe_2_ (with x = 0.1) to approximately 160 K, compared to the T_C_ of ~ 360 K observed in pristine Fe_3_GaTe_2_ [32].

It is noteworthy that electron doping in Fe_3_GaTe_2_ is theoretically predicted to enhance the T_C_ due to the associated increase in magnetic anisotropy energy (MAE) and exchange coupling, as suggested by DFT calculations [32]. A similar trend has been observed in the Fe_n_GeTe_2_ (*n* = 3, 4, 5) material system, where an increase in T_C_ is correlated with higher electron doping levels as n increases [38]. Despite this, Ni doping at the Fe site in Fe_3_GaTe_2_, which serves as an electron donor, leads to a decrease in T_C_ - an outcome contrary to expectations. This unexpected result may be attributed to structural disorder and Fe deficiency induced by Ni doping which reduced MAE [37, 39].

Reducing the thickness of the (Fe_1 − x_Ni_x_)_3_GaTe_2_ (with x = 0.1) flake to 50 nm still preserves the ferromagnetic phase, with a slightly decreased T_C_ of approximately 140 K (Fig. 3c). The T_C_ for the exfoliated flake was extracted by fitting the *M*-*T* data (red solid line in Fig. 3c) according to Eq. (1). The critical exponent *β* for the (Fe_1 − x_Ni_x_)_3_GaTe_2_ (with x = 0.1) flake is 0.334, confirming that the material remains within the three-dimensional ferromagnetic regime even at a thickness of 50 nm [29]. Preserving the bulk T_C_ without a significant reduction as the thickness decreases down to the film regime is a significant advantage for thin-film device applications. This characteristic ensures that the material’s ferromagnetic properties remain robust even at reduced dimensions, which is crucial for the scalability and performance of nanoscale devices. Such stability allows for the continued use of the material in advanced spintronic devices and other applications where maintaining high magnetic ordering at smaller scales is essential.

In both the bulk and 50 nm flake, 10% Ni doping reduces magnetic coercivity (*H*_*c*_), and the square-shaped hysteresis loop becomes rounded (Fig. 3b), indicating a crossover from a ferromagnetic state to a spin glass state, although (Fe_1 − x_Ni_x_)_3_GaTe_2_ (with x = 0.1) still retains its ferromagnetic nature [35, 39].

### Theoretical investigation of dimensionality effect of Fe3GaTe2 material system

**Fig. 4:**
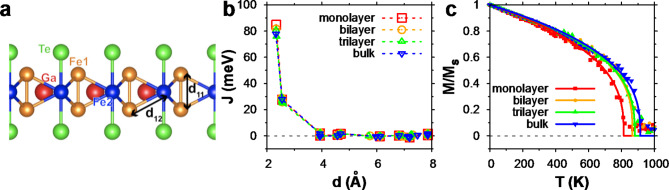
Electronic structures of slabs and bulk Fe_3_GaTe_2_. a, c, e, and g. Electronic band structures of (**a**) monolayer, (**c**) bilayer, (**e**) trilayer, and (**g**) bulk Fe_3_GaTe_2_. b, d, f, and h. The partial density of states of (b) monolayer, (**d**) bilayer, (**f**) trilayer, and (**h**) bulk Fe_3_GaTe_2_

To investigate the relationship between magnetic properties and layer thicknesses, we perform the DFT calculations of monolayer, bilayer, trilayer, and bulk Fe_3_GaTe_2_. Table 1 shows the lattice constants, distances between Fe ions, magnetization, and magnetic moments, calculated with the relaxed atomic positions and lattice constants. The calculated lattice constants are in reasonable agreement with experimental data, with a relative error of about 3–4%. Upon varying the number of layers, the in-plane lattice constant shows almost no change, while the out-of-plane lattice constant *c* (or *2d*_*22,*_ ⊥) exhibits non-monotonic change of less than 2.5% of the *c* lattice constant of the bulk. Moreover, there are no significant differences in the Fe-Fe distances within a layer (see Fig. 4a), as most of the structural changes are in the layer distances. Thus, we find that the structural changes do not induce any significant changes in the magnetization and magnetic moments, consistent with weak vdW interaction between layers. We note that there is a significant difference in the Fe1 and Fe2 magnetic moments also due to the different coordination of Fe ions, as previously reported [40].

**Table 1:** The relaxed structural parameters and magnetic properties of Fe_3_GaTe_2_slabs and bulk. The distances between Fe atoms (see Fig. 4a) are averaging over the corresponding Fe-Fe pairs for all the layers. For the bilayer or trilayer cases, twice the value of the vertical distance of two Fe2 sites between adjacent layers (*2d*_*22,*_ ⊥) is presented instead of the out-of-plane lattice constant *c*. The value of Fe1 and Fe2 magnetic moments are averaged over each layer. The values in the parenthesis for *a* and *c* lattice constants are from experimental data

	a (Å)	c or 2d_22, ⊥_ (Å)	d_11_ (Å)	d_12_ (Å)	M (µ_B_/f.u.)	m_Fe1_ (µ_B_)	m_Fe2_ (µ_B_)
Monolayer	3.931	N/A	2.334	2.552	6.13	2.64	1.60
Bilayer	3.930	16.273	2.328	2.563	6.09	2.60	1.62
Trilayer	3.932	16.178	2.338	2.554	6.07	2.59	1.63
Bulk (Expr.)	3.929 (4.09)	16.584 (16.07)	2.326	2.549	6.09	2.58	1.68

**Fig. 5:**
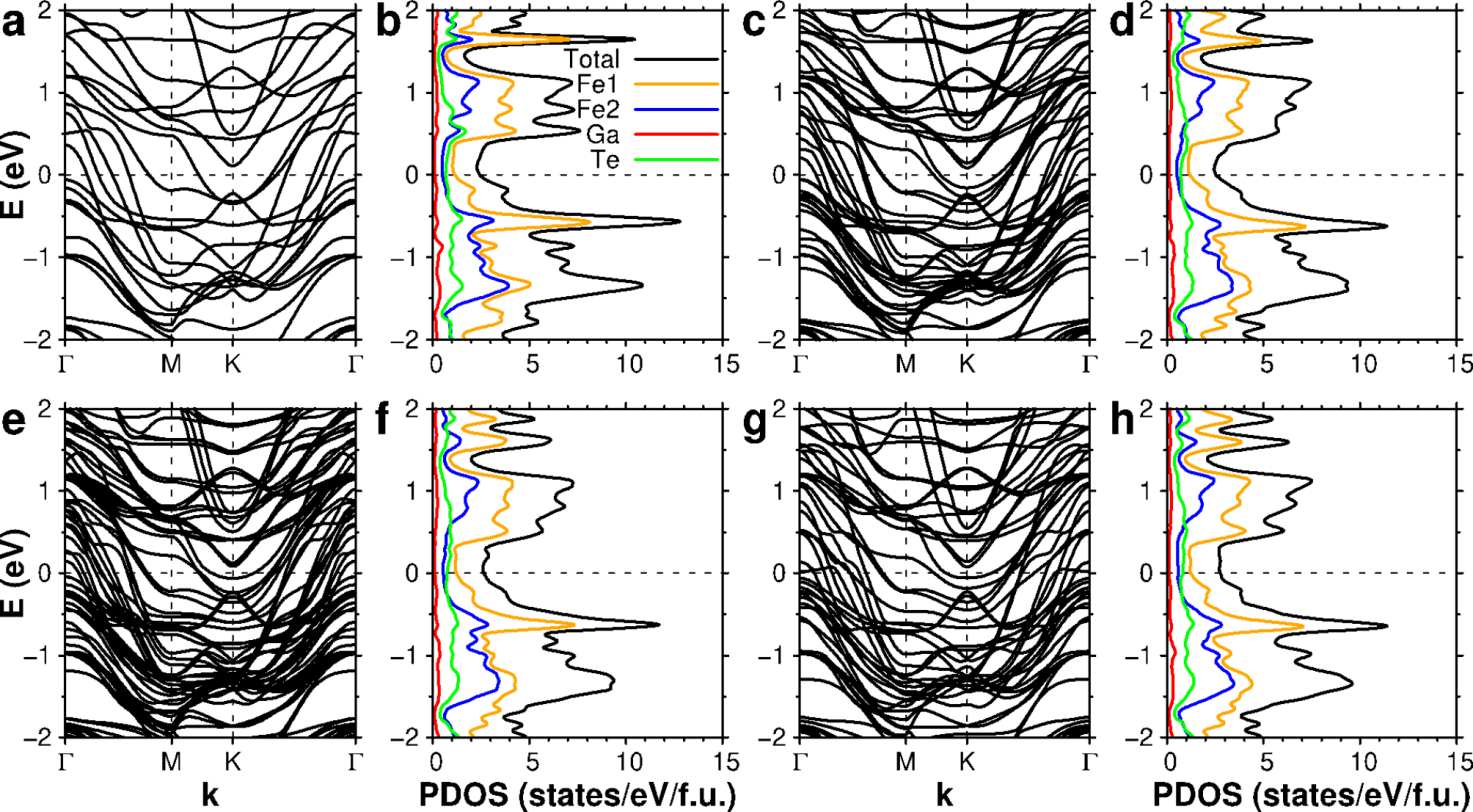
Atomic configurations within a single vdW layer and temperature-dependent magnetic properties Fe_3_GaTe_2_depending on the number of layers. (**a**) Atomic configurations within a layer showing distances between Fe atoms. (**b**) The Heisenberg exchange parameters of monolayer, bilayer, and trilayer structures compared with bulk Fe_3_GaTe_2_ as functions of the distance between Fe sites. (**c**) Magnetization of monolayer, bilayer, trilayer, and bulk Fe_3_GaTe_2_ as a function of temperature. The solid lines are fitted curves using Eq. (1)

Figure 5 presents the comparison of electronic structures with respect to the thickness of Fe_3_GaTe_2_, where we find the partial density of states (PDOS) and band structures do not show significant changes as increasing layer thickness. There are small subband splittings for bilayer and trilayer structures while maintaining overall band energies similar to that of the single layer and bulk cases, consistent with small vdW interlayer interactions. In addition, extra band splitting due to the spin-orbit coupling is present, and the majority of states around the Fermi energy is mainly derived from the majority spin bands (see SM Figure S3). The similarity in the band structures regardless of the number of layers is consistent with magnetization and magnetic moments insensitive to the thickness.

We find a small change in the ferromagnetic transition temperature with increasing layer thickness, consistent with the experimental data. The critical temperatures are calculated using the Heisenberg model in which the exchange parameters between Fe magnetic moments are calculated based on Green’s function method [41]. Figure 4b presents the exchange parameters between Fe ions as a function of Fe-Fe distance where the shortest and the next shortest Fe-Fe distances correspond to **d**_**11**_ and **d**_**12**_ in panel (a), respectively. We find that the exchange parameter decays quickly with respect to the Fe-Fe distances, consistent with previous reports [32, 40, 42], and more importantly, the calculated exchange parameters are insensitive to the layer thickness. This can be understood from the similar band characteristics shared for different Fe_3_GaTe_2_ layers.

Our calculation of T_C_ based on the spin dynamics of the Heisenberg model shows interesting thickness dependence with the T_C_ values of monolayer, bilayer, trilayer, and bulk structures being 814, 867, 881, and 914 K, respectively. We find that there is only a slight increase in T_C_ as the number of layers increases, except for the monolayer case where the calculated transition temperature decreases significantly around 11% of the bulk T_C_. The increase in T_C_ between the trilayer slab and bulk supports our experimental data exhibiting a small increase in T_C_ within the measured thickness range. This can be understood from the weak interlayer vdW couplings, leading to small exchange interactions between adjacent layers. We also expect the same mechanism for Ni-doped cases, as the nature of the interlayer coupling is maintained.

The decrease in T_C_ for the monolayer Fe_3_GaTe_2_ suggests the importance of the small long-range magnetic interaction that can have a significant effect on the T_C_. Although the value of exchange coupling decreases rapidly with the Fe-Fe distance, there exists a long tale of magnetic interactions giving a sizable effect on T_C_, as the numbers of pairs increase with the distance. Our results are consistent with the previous reports showing a large decrease in T_C_ in monolayer and also in line with the significant decrease in T_C_ by truncating the interaction range [40, 43]. We note that our predicted T_C_ values are significantly overestimated, potentially due to the missing dynamic correlation when evaluating exchange parameters within the DFT scheme. The overestimation of T_C_ is also observed in a previous DFT-based study having T_C_ around 600 K for monolayer Fe_3_GaTe_2_ [40]; the difference between our data and previous work could be from the choice of the different exchange-correlation functional (local density approximation (LDA) in Ref [40]. versus generalized gradient approximation (GGA) in our work) due to the tendency of GGA functional tends to enhance magnetism compared with LDA.

## Conclusions

This study investigates the thickness-dependent magnetic properties of Fe_3_GaTe_2_ and (Fe_1 − x_Ni_x_)_3_GaTe_2_ (with x = 0.1) through MOKE measurements and DFT calculations. Our findings reveal that the T_C_ for both Fe_3_GaTe_2_ and (Fe_1 − x_Ni_x_)_3_GaTe_2_ (with x = 0.1) remains relatively stable, exhibiting only a minor decrease as the thickness is reduced to approximately 14 nm and 50 nm, respectively. This result is consistent with our DFT predictions. The ability to maintain ferromagnetic properties at such reduced thicknesses is advantageous for thin-film applications, which is essential for scaling in spintronic devices and the development of next-generation spintronic technologies.

## Methods

### Single crystal growth

Single crystals of Fe_3_GaTe_2_ and (Fe_1 − x_Ni_x_)_3_GaTe_2_ (with x = 0.1) were synthesized by self-flux method. Flakes of Fe (99.98% purity), Ni (99.98% purity), Ga (99.99% purity) and Te (99.99% purity) in the molar ratio were put into a quartz tube. The tube was evacuated and sealed at 0.01 Pa. The sealed quartz ampoule was heated to 1273 K for 10 h and were then held there for another one day, then held there for another one day, then the temperature was quickly decreased down to 1153 K within 2 h followed by slowly cooling down to 1053 K within 100 h. Finally, the ampoule was taken out from the furnace and decanted with a centrifuge to separate (Fe_1 − x_Ni_x_)_3_GaTe_2_ single crystals from the flux. In order to avoid degradation, the (Fe_1 − x_Ni_x_)_3_GaTe_2_ single crystals were stored in an Ar-filled glovebox.

### Flake fabrication

The bulk Fe_3_GaTe_2_ and (Fe_1 − x_Ni_x_)_3_GaTe_2_ (with x = 0.1) samples were stored in an Ar-filled desiccator. These samples were transferred into an Ar-filled glove box (O_2_ / H_2_O concentration = 1.1 ppm/0.7 ppm) and exfoliated onto the wafer using gel packs. A 5 nm Al capping layer was then deposited in-situ using a thermal evaporator (growth rate = 0.3 Å/s, chamber pressure = 8 × 10^− 7^ torr). Immediately after capping, the samples were loaded into the MOKE cryostat to minimize oxidation. Then, cryostat was vacuumed down to 5 × 10^− 6^ torr. This entire process was completed within one day before the MOKE measurement. A schematic of sample preparation system is shown in Figure S1 in the SM.

### Magnetic characterization

Low-temperature magnetic hysteresis loops of the exfoliated Fe_3_GaTe_2_ and (Fe_1 − x_Ni_x_)_3_GaTe_2_ (with x = 0.1) flakes were measured using a polar (out-of-plane) MOKE system. The MOKE system uses a 408 nm diode laser with a laser spot size of ~ 2 microns and has Kerr rotation detection sensitivity < 0.1 mrad.

### First-principles calculations

First-principles DFT calculations were performed using the Perder-Burke-Ernzerhof type generalized gradient approximation [44], norm-conserving pseudopotentials [45], and localized pseudo-atomic orbitals for the wavefunction expansion as implemented in the SIESTA code [46]. The spin-orbit interaction is considered using fully relativistic *j*-dependent pseudopotentials [47] in the *l*-dependent fully separable nonlocal form using additional Kleinman-Bylander-type projectors [48]. For the atomic structure optimization, we use 64 × 64 × 1 k-point mesh for monolayer, bilayer, and trilayer slabs, and 64 × 64 × 16 k-mesh for bulk. Real-space mesh cut-off of 500 Ry is used for all of our calculations. The vdW interaction is evaluated using the DFT-D2 correction [49]. Dipole corrections are included and the vacuum of more than 50 Å is used for the slab calculations to reduce the fictitious interactions between layers generated by the periodic boundary condition in our supercell approach. For self-consistent calculations, 128 × 128 × 1 and 128 × 128 × 32 k-grids are used for the slabs and bulk, respectively. The force theorem [50] is used to calculate the magnetic anisotropic energy. The magnetic exchange parameters are calculated by Green’s function method as implemented in the TB2J package within a 32 × 32 × 1 supercell for slabs and a 32 × 32 × 4 supercell for bulk [41]. The spin dynamics were simulated using the Heun integration scheme as implemented in VAMPIRE [51].

## Electronic supplementary material

Below is the link to the electronic supplementary material.

**Supplementary Material 1**: Flake fabrication of Fe_1 − x_Ni_x_)_3_GaTe_2_ and (Fe1-xNix)3GaTe2 (with x=0.1) single crystals (**Figure S1**). Thickness dependence of magnetic properties of Fe_1 − x_Ni_x_)_3_GaTe_2_ flakes (**Figure S2**). Electronic structures of Fe_1 − x_Ni_x_)_3_GaTe_2_ without spin-orbit coupling (**Figure S3**).

## Data Availability

The datasets used and/or analyzed during the current study are available from the corresponding author on reasonable request.

## Abbreviations

AI: Artificial intelligence
AR: Augmented reality
IoT: Internet of things
ReRAM: Resistive random access memory
PCRAM: Phase-change random-access memory
HBM: High bandwidth memory
CXL: Compute express link
PIM: Processing in memory
CDW: Charge density waves
MOKE: Magneto-optical Kerr effect
DFT: Density functional theory
AFM: Atomic force microscopy
SM: Supplementary materials
M_S_: Saturation magnetization
ZFC: Zero-field cooling
FC: Field cooling
MAE: Magnetic anisotropy energy
H_C_: Magnetic coercivity
PDOS: Partial density of states
LDA: Local density approximation
GGA: Generalized gradient approximation
