# Correction: Persistent ferromagnetic ground state in pristine and Ni-doped Fe_3_GaTe_2_ flakes

**DOI:** 10.1186/s40580-024-00471-0

**Published:** 2025-02-25

**Authors:** Ki-Hoon Son, Sehoon Oh, Junho Lee, Sobin Yun, Yunseo Shin, Shaohua Yan, Chaun Jang, Hong-Sub Lee, Hechang Lei, Se Young Park, Hyejin Ryu

**Affiliations:** 1 Center for Semiconductor Technology, Korea Institute of Science and Technology (KIST), Seoul, 02792 South Korea; 2 Department of Advanced Materials Engineering for Information and Electronics, Kyung Hee University, Yongin, 17104 South Korea; 3 Department of Physics and Origin of Matter and Evolution of Galaxies (OMEG) Institute, Soongsil University, Seoul, 06978 South Korea; 4 Integrative Institute of Basic Sciences, Soongsil University, Seoul, 06978 South Korea; 5 School of Physics and Beiing Key Laboratory of Optoelectronic Functional Materials MicroNano Devices, Renmin University of China, Beijing, 100872 China; 6 Key Laboratory of Quantum State Construction and Manipulation (Ministry of Education), Renmin University of China, Beijing, 100872 China

**Correction to: Nano Convergence (2024) 11:55** 10.1186/s40580-024-00458-x

The original publication of this article [1] was inadvertently published without graphical abstract which should have appeared as shown below.

## Graphical Abstract



In section 2.3, the ferromagnetic properties should be “Fe_3_GaTe_2_” instead of “Fe3GaTe2”. The correct section heading should be “2.3 Theoretical investigation of dimensionality effect of Fe_3_GaTe_2_ material system”.

The captions and the images of Figures 4 and 5 were interchanged in the original publication of the article. Figures 4 and 5 are presented with the corresponding captions in this correction article.

**Fig. 4:**
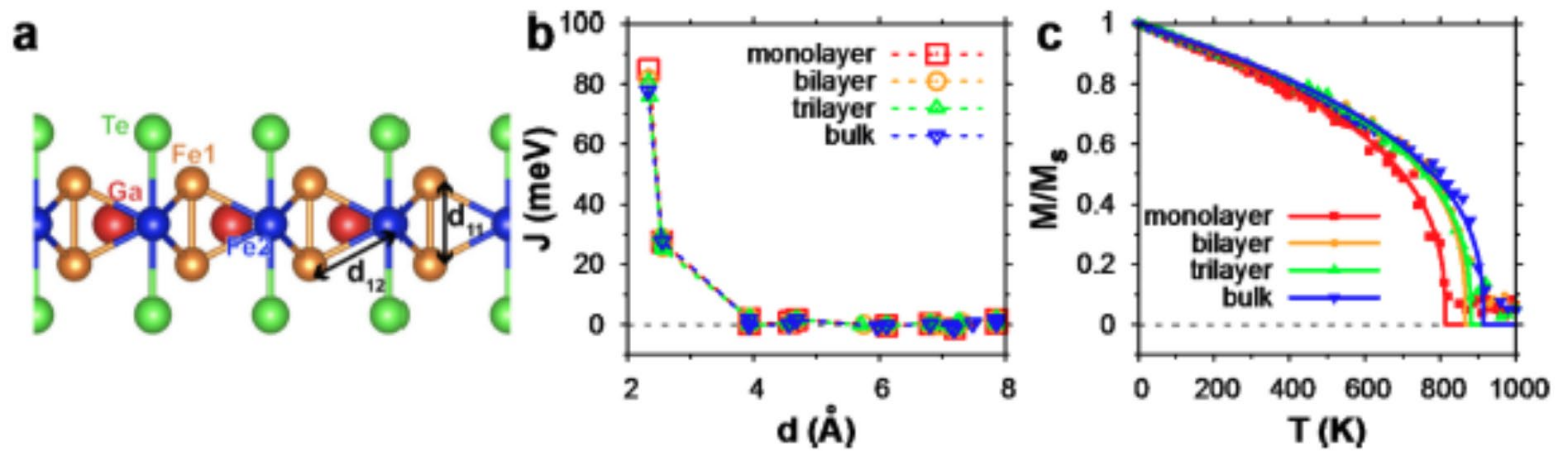
Atomic configurations within a single vdW layer and temperature-dependent magnetic properties Fe_3_GaTe_2_ depending on the number of layers. (a) Atomic configurations within a layer showing distances between Fe atoms. (b) The Heisenberg exchange parameters of monolayer, bilayer, and trilayer structures compared with bulk Fe_3_GaTe_2_ as functions of the distance between Fe sites. (c) Magnetization of monolayer, bilayer, trilayer, and bulk Fe_3_GaTe_2_ as a function of temperature. The solid lines are fitted curves using Eq. (1)

**Fig. 5:**
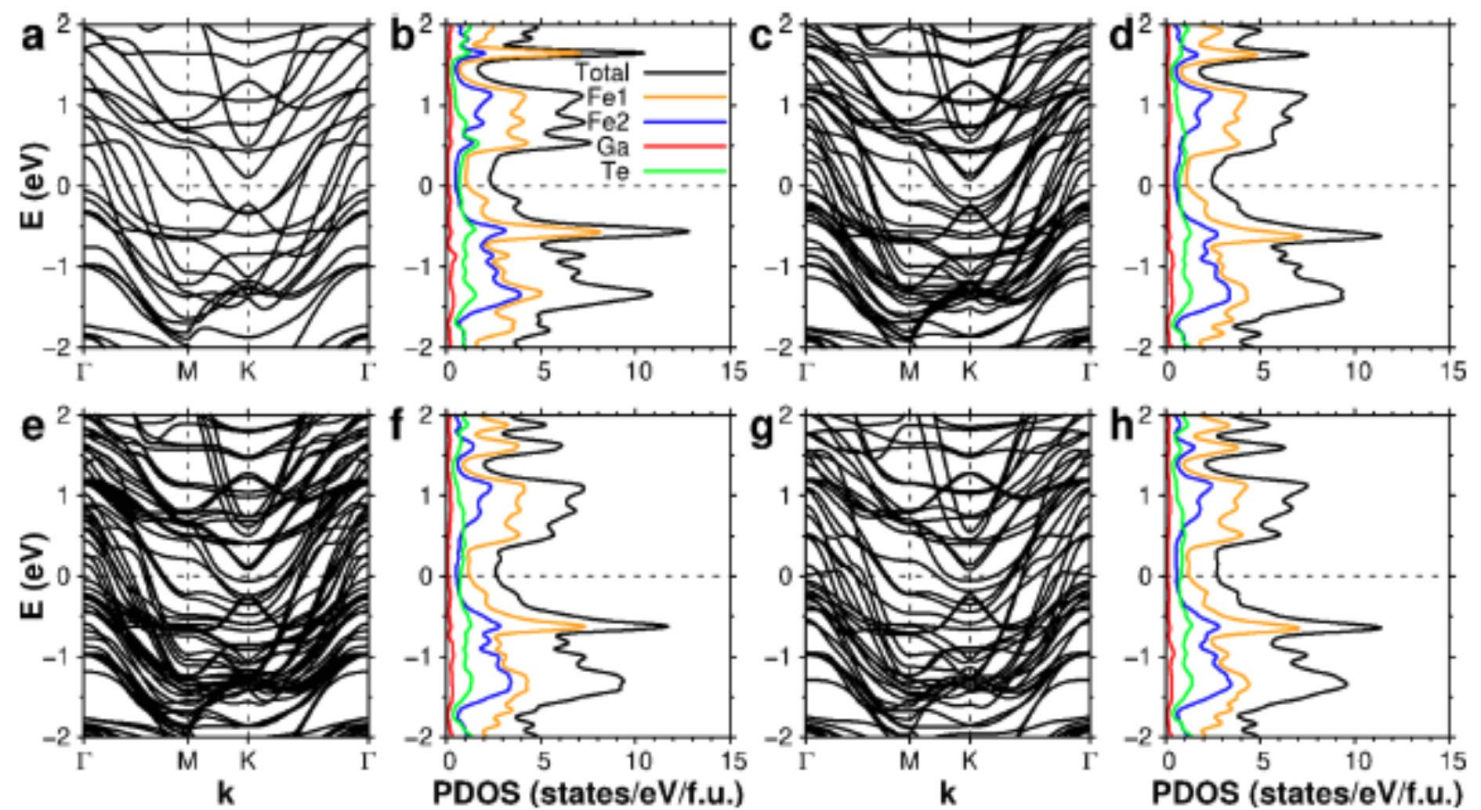
Electronic structures of slabs and bulk Fe_3_GaTe_2_. **a**, **c**, **e**, and **g**. Electronic band structures of (**a**) monolayer, (**c**) bilayer, (**e**) trilayer, and (**g**) bulk Fe_3_GaTe_2_. **b**, **d**, **f**, and **h**. The partial density of states of (**b**) monolayer, (**d**) bilayer, (**f**) trilayer, and (**h**) bulk Fe_3_GaTe_2_
