# Supplementary material for: Persistent ferromagnetic ground state in pristine and Ni-doped Fe3GaTe2 flakes
Source: Nano Converg. 2024 Dec 12;11:55. doi: 10.1186/s40580-024-00458-x (PMC11638437; doi:10.1186/s40580-024-00458-x)
Supplement: Supplementary file 1 — Supplementary Material 1: Flake fabrication of Fe1 − xNix)3GaTe2 and (Fe1-xNix)3GaTe2 (with x=0.1) single crystals (Figure S1). Thickness dependence of magnetic properties of Fe1 − xNix)3GaTe2 flakes (Figure S2). Electronic structures of Fe1 − xNix)3GaTe2 without spin-orbit coupling (Figure S3). [file 40580_2024_458_MOESM1_ESM.docx]

**Supplementary Materials for**

**Persistent Ferromagnetic Ground State in Pristine and Ni-Doped Fe_3_GaTe_2_ Flakes**

Ki-Hoon Son^1,2,†^, Sehoon Oh^3,4,†^, Junho Lee^1^, Sobin Yun^1^, Yunseo Shin^2^, Shaohua Yan^5,6^, Chaun Jang^1^, Hong-Sub Lee^2,*^, Hechang Lei^5,6,*^, Se Young Park^3,4 *^, and Hyejin Ryu^1,*^

*^1^Center for Semiconductor Technology, Korea Institute of Science and Technology (KIST), Seoul 02792, South Korea*

*^2^Department of Advanced Materials Engineering for Information and Electronics, Kyung Hee University, Yongin 17104, South Korea*

*^3^Department of Physics and Origin of Matter and Evolution of Galaxies (OMEG) Institute, Soongsil University, Seoul 06978, South Korea*

*^4^Integrative Institute of Basic Sciences, Soongsil University, Seoul, 06978, South Korea*

*^5^School of Physics and Beiing Key Laboratory of Optoelectronic Functional Materials MicroNano Devices, Renmin University of China, Beijing 100872, China*

*^6^Key Laboratory of Quantum State Construction and Manipulation (Ministry of Education), Renmin University of China, Beijing, 100872, China*

**Flake fabrication of Fe_3_GaTe_2_ and (Fe_1-x_Ni_x_)_3_GaTe_2_ (with x=0.1) single crystals**


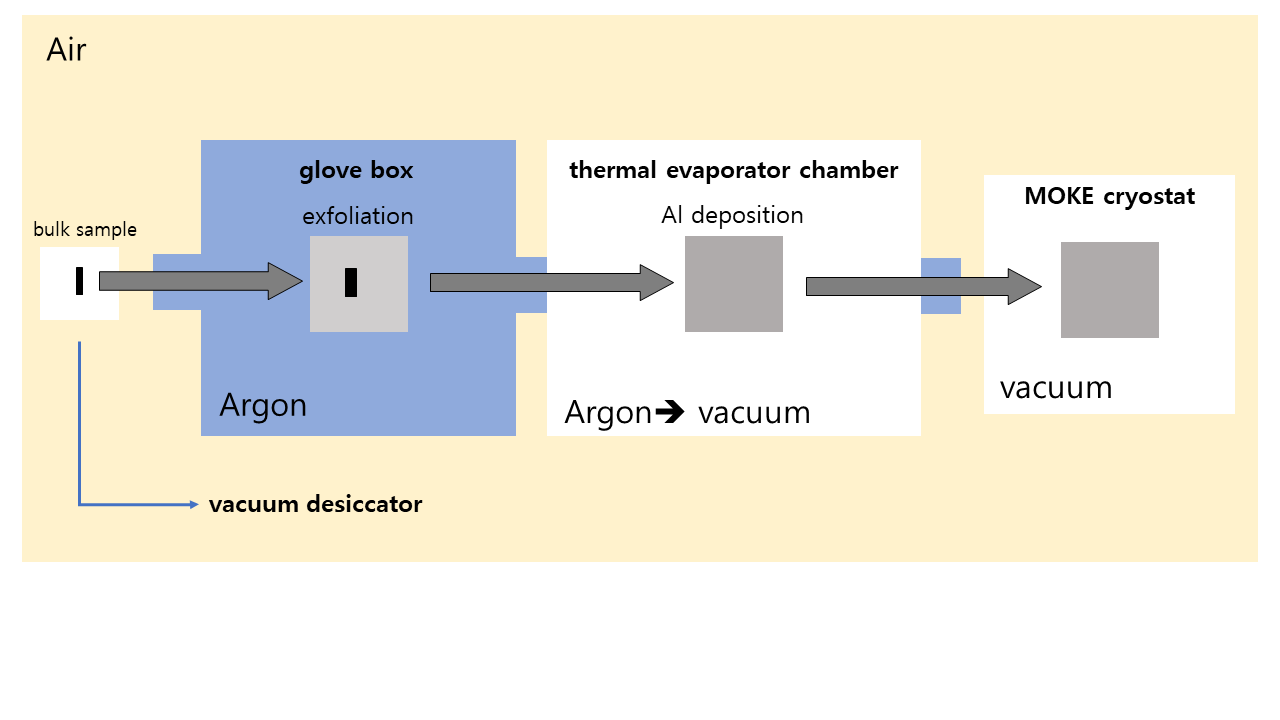


**Figure S1. A schematic diagram of flake fabrication system.** The protocol implemented to prevent sample oxidation. The glove box and thermal evaporator chamber are connected *in situ*.

**Thickness dependence of magnetic properties of Fe_3_GaTe_2_ flakes**

**
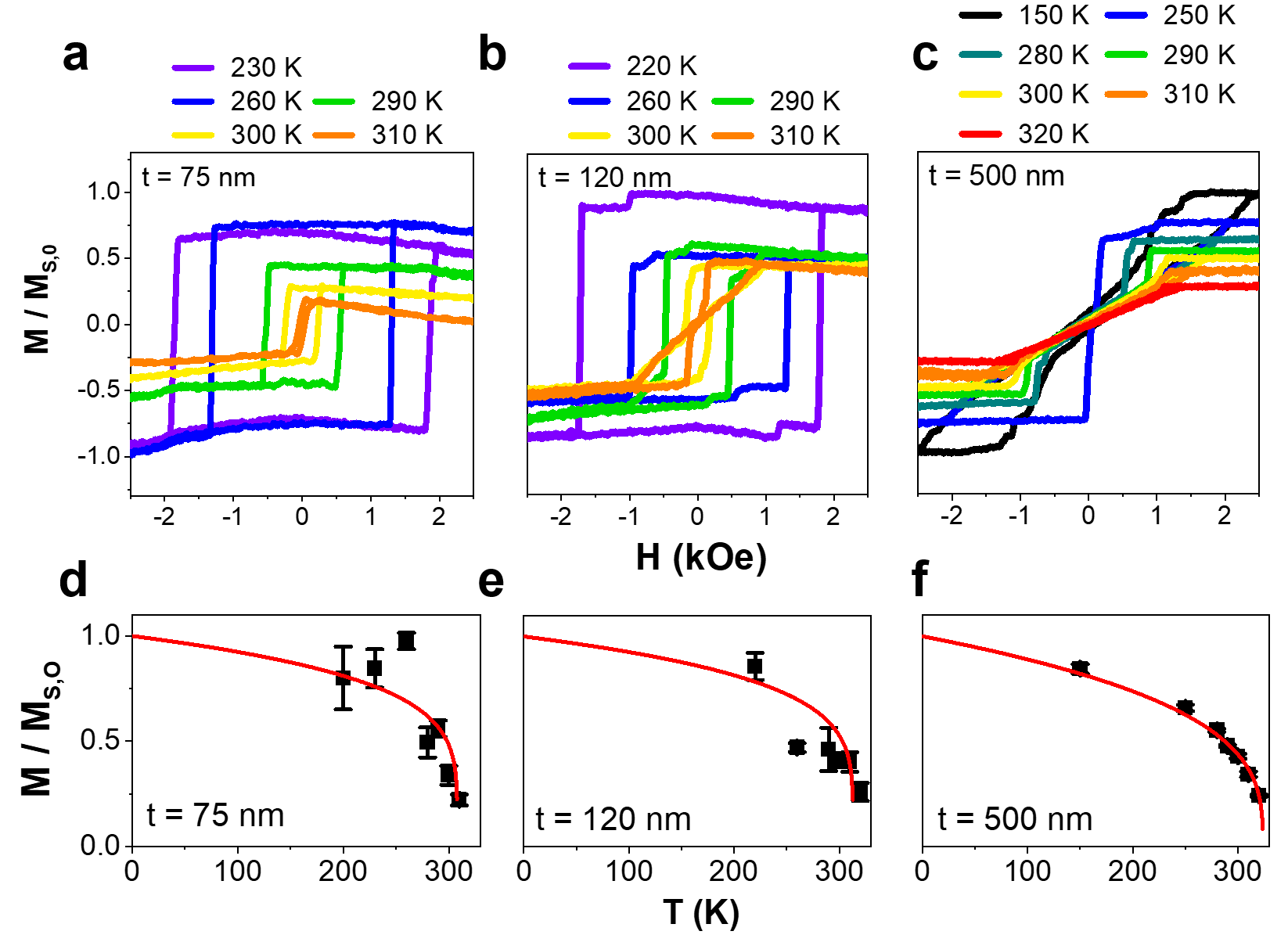
**

**Figure S2. Thickness dependence of magnetic properties of pristine Fe_3_GaTe_2_ flakes.** **a-c.** Temperature-dependent out-of-plane M-H loops of Fe_3_GaTe_2_ flakes measured by MOKE system at several thicknesses (t). The colors uniformly represent each temperature. In the case of **c**, the vanishing remanence is due to the formation of magnetic domains in the thick Fe_3_GaTe_2_ flakes, as the dipolar interaction increases with thickness. **d-f.** Temperature-dependent Ms extracted from **a**-**c** along with the fitting curves (red solid lines). The magnetizations in **a**-**f** are normalized by the extrapolated M_S_ at T = 0 (M_S,0_) obtained from the fitting in **d**-**f**.

**Electronic structures of Fe_3_GaTe_2_ without spin-orbit coupling**

**Figure S3. Electronic structures of slabs and bulk Fe_3_GaTe_2_ in the absence of spin-orbit coupling.** **a,c,e** and **g.** Electronic band structures of **a** monolayer, **c** bilayer, **e** trilayer, and **g** bulk Fe_3_GaTe_2_. **b,d,f** and **h.** The partial density of states of **b** monolayer, **d** bilayer, **f** trilayer, and **h** bulk Fe_3_GaTe_2_.
